# Natural frequencies improve Bayesian reasoning in simple and complex inference tasks

**DOI:** 10.3389/fpsyg.2015.01473

**Published:** 2015-10-14

**Authors:** Ulrich Hoffrage, Stefan Krauss, Laura Martignon, Gerd Gigerenzer

**Affiliations:** ^1^Faculty of Business and Economics (HEC Lausanne), University of LausanneLausanne, Switzerland; ^2^Mathematics Education, Faculty of Mathematics, University of RegensburgRegensburg, Germany; ^3^Institute of Mathematics, Ludwigsburg University of EducationLudwigsburg, Germany; ^4^Center for Adaptive Behavior and Cognition, Max Planck Institute for Human DevelopmentBerlin, Germany

**Keywords:** Bayesian inference, representation of information, natural frequencies, task complexity, instruction, fast-and-frugal trees, visualization

## Abstract

Representing statistical information in terms of natural frequencies rather than probabilities improves performance in Bayesian inference tasks. This beneficial effect of natural frequencies has been demonstrated in a variety of applied domains such as medicine, law, and education. Yet all the research and applications so far have been limited to situations where one dichotomous cue is used to infer which of two hypotheses is true. Real-life applications, however, often involve situations where cues (e.g., medical tests) have more than one value, where more than two hypotheses (e.g., diseases) are considered, or where more than one cue is available. In Study 1, we show that natural frequencies, compared to information stated in terms of probabilities, consistently increase the proportion of Bayesian inferences made by medical students in four conditions—three cue values, three hypotheses, two cues, or three cues—by an average of 37 percentage points. In Study 2, we show that teaching natural frequencies for simple tasks with one dichotomous cue and two hypotheses leads to a transfer of learning to complex tasks with three cue values and two cues, with a proportion of 40 and 81% correct inferences, respectively. Thus, natural frequencies facilitate Bayesian reasoning in a much broader class of situations than previously thought.

## Introduction

After a positive hemoccult screening test, which signals hidden blood in the stool, a patient asks his doctor: “What does a positive result mean? Do I definitely have colon cancer? If not, how likely is it?” When 24 experienced physicians, including heads of departments, were asked this, their answers to the third question ranged between 1 and 99% (Hoffrage and Gigerenzer, 1998). All these physicians had the same information: a prevalence of 0.3%, a sensitivity of 50%, and a false positive rate of 3%. Bayes' rule shows that the actual probability of colon cancer given a positive result is about 5%. As this and subsequent studies have documented, most physicians do not know how to estimate the probability of cancer given the prevalence, sensitivity, and false positive rate of a test (Gigerenzer, 2014). This difficulty has also been observed in laypeople and attributed to some internal mental flaw, such as a general base rate neglect, the representative heuristic, or a general inability to reason the Bayesian way (e.g., Kahneman, 2011). Yet the experimental evidence has made it clear that the problem is not simply in our minds, but in the way the information is presented. When Hoffrage and Gigerenzer (1998) gave another group of 24 physicians the same information in *natural frequencies* (see below), 16 of these could find the Bayesian answer, namely that a patient actually has cancer in only 1 out of 20 positive screening results. When given *conditional probabilities*, that is, the sensitivity and false alarm rate, only 1 out of 24 physicians could find the Bayesian answer, or anything close to it.

The positive effect of natural frequencies on Bayesian reasoning was first documented by Gigerenzer and Hoffrage (1995, 1999) and has since been confirmed in both numerous laboratory studies (e.g., Cosmides and Tooby, 1996; Brase, 2002, 2008) and applied research, including screening for Down syndrome (Bramwell et al., 2006), the interpretation of DNA evidence in court (Lindsey et al., 2003), and teaching children to reason the Bayesian way (Zhu and Gigerenzer, 2006). Thus, the earlier claim that people's cognitive limitations make them poor Bayesians (e.g., Kahneman and Tversky, 1972, repeated in Kahneman, 2011, and Thaler and Sunstein, 2008) is now known to be incorrect; it holds only when information is presented in probabilities. When presented in natural frequencies, by contrast, Bayesian performance increases substantially.

Yet there is a limitation to virtually all of these studies. Whether using conditional probabilities or natural frequencies, the experimental studies that have been conducted so far incorporated solely the simplest version of a Bayesian task—henceforth referred to as the *basic task*—which involves two hypotheses (such as colon cancer or no colon cancer) and a single cue (such as the hemoccult test) with two cue values (a positive or negative result). In 1998, Massaro questioned whether the facilitating effect of natural frequencies extends to more complex tasks that involve two or more cues. He conjectured that even in the case of two cues, “a frequency algorithm will not work” (p. 178). Although he did not test this claim, if true, it would severely limit the range of applications of natural frequencies. In this article, we experimentally test Massaro's claim, as well as whether the effect of natural frequencies generalizes to tasks involving three cues, three cue values, and three hypotheses.

This article has two parts. In the first, we outline the two paradigms for studying Bayesian reasoning, which use two different methodologies and have arrived at apparently contradicting conclusions concerning people's ability to reason the Bayesian way. One is a learning paradigm where probabilities are learned by sequentially observing events; the other is the classical textbook paradigm where people are assigned problems with specified conditional probabilities. We show that natural frequency representations are a kind of missing link between the two paradigms. In the second part, we report two studies. The first study tests whether the beneficial effect of natural frequencies generalizes to more complex Bayesian inferences, that is, to tasks containing more than two hypotheses, more than one cue, or cues with more than two values. The second study tests whether a short instruction in natural frequencies for a basic task (involving one dichotomous cue and two hypotheses) facilitates applying Bayesian reasoning to complex tasks. In the discussion we relate the present work to the fast-and-frugal heuristics program and to other interventions to boost performance in Bayesian inference tasks.

## Paradigms to study bayesian inferences: probability learning and textbook tasks

A Bayesian inference task is a task in which the probability *p*(*H*|*D*) of some hypothesis *H* (e.g., cancer) given data *D* (e.g., a test result) has to be estimated. Two types of Bayesian inference tasks can be distinguished (Gigerenzer, 2015; Mandel, 2015; Sirota et al., 2015b): probability learning and textbook tasks.

Let us first consider probability learning tasks. Organisms learn the consequences of various behavioral responses in a probabilistic environment with multiple cues. Note that such a task ultimately requires behavioral responses in a specific situation. For instance, what should a bird do when it sees a movement in the grass? This situation can be conceived as a Bayesian inference task in which the behavioral response is based on a comparison of the probability that the movement of the grass (data, *D*) is caused by something that is dangerous (hypothesis, *H*) or by something that is not dangerous (–*H*). In the laboratory, a probability learning task involves the sequential encounter of pairs of events. In the case of two hypotheses (*H* and its complement –*H*) and two possible states of the world (data *D* observed or not), there are four possible pairs: *H*&*D, H*&–*D*, –*H*&*D*, –*H*&–*D*. To answer the Bayesian question “what is *p*(*H*|*D*)?” one needs to compare the two possibilities *D*&*H* and *D*&–*H* with respect to their probabilities. How likely is “grass movement due to dangerous cause (e.g., cat)” compared to “grass movement for some other non-dangerous reason (e.g., wind)”? How likely is “hemoccult test positive and patient has colon cancer” compared to “test positive for some other reason”? Transforming the odds of the two possibilities—one probability compared to the other—into a ratio amounts to dividing the first probability by the sum of both:

(1) $$p(H|D)\;=\;\frac{p\left(D\&H\right)}{p\left(D\right)}\;=\;\frac{p\left(D\&H\right)}{p\left(D\&H\right)+p\left(D\&-H\right)}$$

where *p(H|D)* stands for the posterior probability that the hypothesis *H* is true given the observed data *D*. Equation (1) is one form of Bayes' rule.

The probabilities relevant for Bayesian inferences can be learned via three paths: phylogenetic learning (natural selection of inherited instincts, i.e., evolutionary preparedness; Harlow, 1958), ontogenetic learning (e.g., classical and instrumental conditioning; Pearce, 1997), and, for some species, social learning (Richerson and Boyd, 2008). A major conclusion of the probability learning paradigm is that humans and animals are approximate Bayesians (Anderson, 1990; Gallistel, 1990; Chater et al., 2006; Chater and Oaksford, 2008).

Let us now turn to the second type of Bayesian inference tasks, textbook tasks. In their evolutionary history, humans have developed skills that other species have in some rudimentary form, but which humans master at a far superior level: social learning, instruction, and reasoning (Richerson and Boyd, 2008). These skills enable culture, civilization, science, and textbooks. Moreover, they facilitate communication of probabilities, one of the many examples of how ontogenetic learning of probabilities can be supported by social learning (McElreath et al., 2013). Last but not least, they allow for the development of probability theory, which, in turn, offers a formal framework for evaluating hypotheses in light of empirical evidence. Even though the question of how this should be done is an ancient one, only since the Enlightenment have hypotheses been evaluated in terms of mathematical probability (Daston, 1988). Specifically, when evaluating an uncertain claim (i.e., hypothesis), the posterior probability of the claim can be estimated after new data have been obtained. One rigorous method for doing so was established by Thomas Bayes and, later, Pierre Simon de Laplace. The mathematical expression for updating hypotheses in light of new data is given in Equation (2):

(2) $$p(H|D)\;=\;\frac{p\left(H\right)p(D|H)}{p\left(H\right)p\left(D\text{​​}|H\right)\;+\;p(-H)p\left(D\text{​}|-H\right)}$$

where *p(H)* and *p(*–*H)* stand for the prior probabilities that the hypothesis (*H*) and its complement (–*H*), are true, and where *p(D|H)* and *p*(*D*|–*H*) stand for the likelihood of observing the data under these two different conditions. In signal detection theory, these two likelihoods are referred to as hit rate and false-alarm rate. In medical terms, the hit rate is the sensitivity of a diagnostic test and the false-alarm rate is the complement of the specificity of the test. Equation (2) formalizes how prior probabilities and likelihoods should be combined to compute the Bayesian posterior probability. Note that this equation is a variant of Equation (1) in which the two conjunctions, *p(D*&*H*) and *p*(*D*&–*H*), are broken into components. Strictly speaking, Equation (1), albeit a form of Bayes' rule, is not an equation that captures the updating of probabilities. Unlike Equation (2), Equation (1) does not describe the relationship between *p(H)* and *p(H|D)*, simply because it does not include the term *p(H)*.

Social learning, probability theory, and Bayes' rule in the form of Equation (2) offer a new opportunity: to study Bayesian reasoning using textbook tasks with specified probabilities that do not need to be learned from experience. In contrast to the probability learning paradigm with its sequential input of observations, the textbook paradigm provides the information as a final tally (usually in numerical form). Whereas the most important cognitive ability required to solve a Bayesian task in the probability learning paradigm is frequency encoding (and memory), the most useful cognitive abilities in the textbook task paradigm are reasoning and calculation (for a discussion of Bayesian reasoning in textbox tasks adopting a problem-solving approach, see Johnson and Tubau, 2015). Note that the distinction between (Bayesian) behavior in the context of the probability learning paradigm and (Bayesian) reasoning in the context of the textbook paradigm is akin to Hertwig et al.'s (2004) distinction between decisions-from-experience and decisions-from-descriptions. But there are two kinds of descriptions within the textbook task paradigm: The statistical information can be presented in terms of either conditional probabilities or natural frequencies, which, as the introductory example illustrated, has quite opposite effects on reasoning.

## Performance in bayesian textbook problem solving depends on the representation format

It is striking to see the differences obtained by the two research paradigms (Gigerenzer, 2015; Mandel, 2015; Sirota et al., 2015b). Whereas the probability learning paradigm depicts humans and animals as approximate Bayesians (at least in the simple tasks studied), early research using the textbook paradigm arrived at a different conclusion. This discrepancy went mostly unnoticed because cross-references between the researchers in both paradigms have been rare. In their introductory note to the present special issue, Navarrete and Mandel (2015) distinguish three waves in the history of this research using the textbook paradigm. The first wave was marked by Edwards (1968) with his urns-and-balls problems. In the vignettes of these problems, prior probabilities [i.e., *p*(*H*) and *p*(–*H*)] were communicated but no likelihoods [i.e., *p*(*D*|*H*) and *p*(*D*|–*H*)]—although the sample information that was given instead (e.g., 4 blue balls and 1 red ball) potentially allowed for calculating the corresponding likelihoods. Edwards (1968) found that if people have to update their opinions, they change their view in the direction proposed by Bayes' rule. However, he also reported that people are “conservative Bayesians” in the sense that they do not update their prior beliefs as strongly as required by Bayes' rule.

A study by Eddy (1982) illustrates the second wave of research. The question he asked was: Do experts reason the Bayesian way? Eddy found that physicians' judgments did not follow Bayes' rule when solving the following type of task (a prototypical Bayesian situation):

The probability of breast cancer is 1% for a woman at age 40 who participates in routine screening. If a woman has breast cancer, the probability is 80% that she will get a positive mammography. If a woman does not have breast cancer, the probability is 9.6% that she will also get a positive mammography. A woman in this age group had a positive mammography in a routine screening. What is the probability that she actually has breast cancer?

According to Bayes's rule, the answer is 7.8%, which can be obtained by inserting the given information into Equation (2). Yet Eddy (1982) reported that 95 out of 100 physicians estimated this probability to be between 70 and 80%. He argued that these physicians confused the conditional probability of breast cancer given a positive mammogram with that of a positive mammogram given breast cancer. To explain the failure of Bayesian reasoning, Kahneman and Tversky (1972) suggested the “representativeness heuristic,” although it remains unclear whether the heuristic concurs with Eddy's explanation because this “one-word explanation” (Gigerenzer, 1996, p. 594) has never been defined and formalized (see Gigerenzer and Murray, 1987). Be that as it may, Kahneman and Tversky (1972) concluded: “In his evaluation of evidence man is apparently not a conservative Bayesian: he is not Bayesian at all” (p. 450).

Whereas the second wave attributed failure in Bayesian reasoning to flawed mental processes, a third wave starting in the mid-1990s (Gigerenzer and Hoffrage, 1995, 1999; Cosmides and Tooby, 1996) showed experimentally that much of the problem lies in how risk is represented. Specifically, Gigerenzer and Hoffrage established that it is not Bayesian reasoning *per se* that is difficult but rather the format of information provided to the participants. In Eddy's (1982) task, quantitative information was provided in conditional probabilities. Gigerenzer and Hoffrage (1995) showed that such a representation format makes the computation of the Bayesian posterior probability more complicated than with natural frequencies. Natural frequencies result from natural sampling and have historically been the “natural” input format for the human mind (Kleiter, 1994; Gigerenzer and Hoffrage, 1999, pp. 425–426). Presenting the information in Eddy's mammography task in terms of natural frequencies yields the following description:

10 out of every 1000 women at age 40 who participate in routine screening have breast cancer. 8 out of every 10 women with breast cancer will get a positive mammography. 95 out of every 990 women without breast cancer will also get a positive mammography. Here is a new representative sample of women at age 40 who got a positive mammography in a routine screening. How many of these women do you expect to actually have breast cancer?

Answering this question amounts to solving Equation (3):

(3) $$p(H|D)=\;\frac{f\left(D\&H\right)}{f(D)}=\;\frac{f\left(D\&H\right)}{f\left(D\&H\right)+f\left(D\&-H\right)}$$

where *f(D*&*H)* stands for the natural frequency of joint occurrences of *D* and *H, f(D*&–*H)* stands for the natural frequency of joint occurrences of *D* and –*H*, and *f(D)* for their sum. In the mammography problem, these two joint occurrences are 8 and 95 (out of 1000 women), respectively, and hence there are, in sum, 103 women who get a positive mammogram. Of 103 women who get a positive mammogram, 8 actually have breast cancer. This relative frequency of 8/103 corresponds to a posterior probability of 7.8%, the number that we already computed using Equation (2). Note that natural frequencies result from drawing N objects (e.g., 1000 in the above example) at random from a larger population (or from taking the entire population). Any decomposition of this sample of size N contains natural frequencies, which can be interpreted only in relation to each other and in relation to the total sample size N. Attempts to illustrate what natural frequencies are by simply naming “1 of 10” as an example and in isolation from any other number of a natural frequency tree misses this important point.

When information has been presented in terms of natural frequencies, almost half of Gigerenzer and Hoffrage's (1995) student participants found the Bayesian answer. Among 160 gynecologists, the proportion of Bayesian answers increased from 21 to 87% for probabilities and natural frequencies, respectively (Gigerenzer et al., 2007). The beneficial effect of natural frequency representations has been replicated with experienced physicians (Hoffrage and Gigerenzer, 1998; Bramwell et al., 2006), patients (Garcia-Retamero and Hoffrage, 2013), judges (Hoffrage et al., 2000), and managers (Hoffrage et al., 2015), and has been used to design tutorials on Bayesian reasoning (Sedlmeier and Gigerenzer, 2001; Kurzenhäuser and Hoffrage, 2002).

Textbook problems with information provided in terms of natural frequencies are in fact close to the probability learning paradigm [see the similarity between Equations (1) and (3)]. In contrast, textbook problems with information provided in terms of probabilities do not bear much resemblance to this paradigm [note the difference between Equation (2), with its three pieces of information, and Equation (1), with its two pieces of information]. Natural frequencies are related to the probability learning paradigm because they are the final tally that result from what has been called “natural sampling” (Kleiter, 1994) which, in turn, can be conceived as the process of sequentially observing one event after the other in a natural environment. In other words, natural sampling is the process underlying experiential learning—the paradigm in which humans and animals tend to perform well (Hasher and Zacks, 1979; Gallistel, 1990). Thus, it is no surprise that the beneficial effect of natural frequency representations could be found even for 4th and 5th graders (Zhu and Gigerenzer, 2006; Gigerenzer, 2014; Multmeier, unpublished manuscript; see also Till, 2013). The comparison between Equations (2) and (3) shows why natural frequencies facilitate Bayesian inference. It simplifies computation of the posterior probability: The representation does part of the computation (Gigerenzer and Hoffrage, 2007; Hill and Brase, 2012; Brase and Hill, 2015).

In subsequent work, the power of representation formats has been discussed in a wider context that also embraces important issues such as trust, transparency, or institutional design, to name a few (see Gigerenzer, 2002, 2014; Gigerenzer et al., 2007; Gigerenzer and Gray, 2011). As a consequence of all this research, of various activities to propagate it, and of the desire and pressure to improve Bayesian inference in several domains, the use of natural frequencies is recommended by major evidence-based medical societies, including the Cochrane Collaboration (Rosenbaum et al., 2010), the International Patient Decision Aid Standards Collaboration (Trevena et al., 2012), the Medicine and Healthcare Products Regulatory Agency (the United Kingdom's equivalent to the Food and Drug Administration; see Woloshin and Schwartz, 2011), and the Royal College of Obstetricians and Gynecologists (2008). Moreover, natural frequencies are used in some of the most important school textbooks and in textbooks for future teachers of stochastics in school in the German speaking countries (Martignon, 2011), and they are already part of the school syllabus in the United Kingdom (Spiegelhalter and Gage, 2014).

Yet, as mentioned in the introduction, these developments are severely limited by the fact that up to now, the studies on which they are based used only simple versions of Bayesian tasks with one dichotomous cue and two hypotheses.

## Types of bayesian inference tasks: the basic task and complex tasks

**Figure 1:**
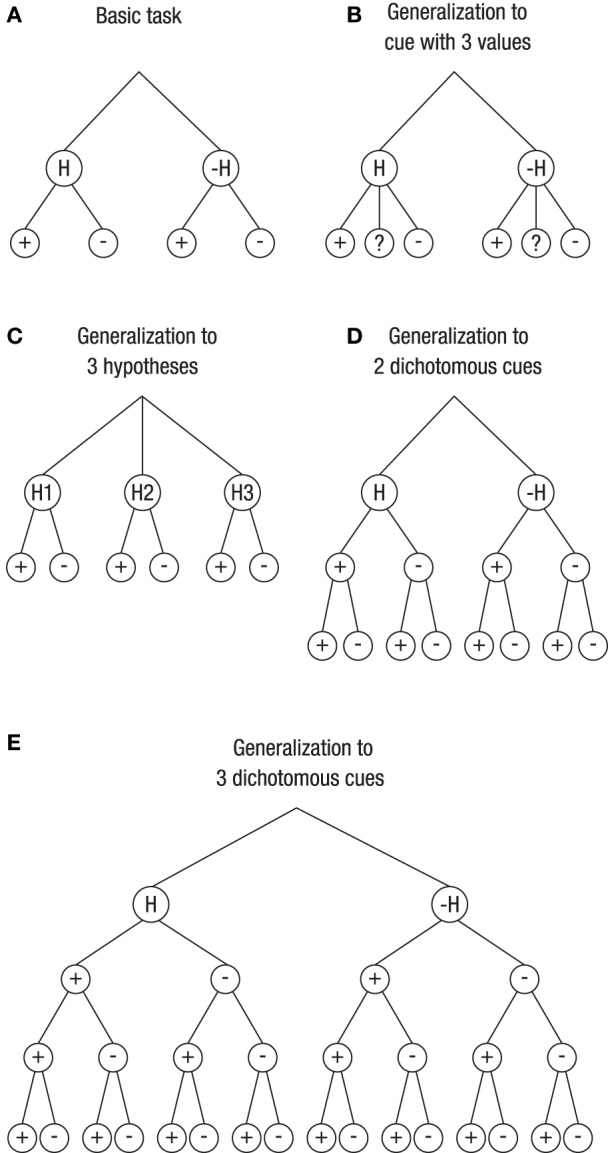
**Generalization of the basic Bayesian inference task (with two hypotheses and one dichotomous cue; A) to more complex tasks (B–E)**. The layers below the hypotheses depict the cue values (or data). Unknown cue values are denoted as “?” **(B)**. For a pair of two hypotheses (one being the complement of the other), these are denoted as H and –H **(A,B,D,E)**, and for a triple of hypotheses, they are denoted as H1, H2, and H3 **(C)**.

There is one important difference between real-life probability learning tasks and textbook problem solving. Compared to most real-life situations, the textbook problems in the literature on Bayesian reasoning are relatively simple. The vast majority of them involve two hypotheses and one dichotomous cue. As mentioned before, we refer to such a task as a basic task. Many real-life situations, in contrast, are more complex. We see three ways in which the basic task can be extended; Figure 1 depicts the basic task (Figure 1A) and these extensions (Figures 1B–E).

One extension involves a situation with a cue having more than two levels (Figure 1B). In fact, many variables are polychotomous. Others may even be continuous and have been divided, for various reasons, into several categories by using cutoffs. For instance, mammograms obtained in a screening program are not simply positive or negative but depict breast cancers that vary in size, shape, or density, the fact of which led to the BI-RADS classification that distinguishes multiple categories. Generally speaking, for polychotomous cues, there is not only one hit rate, *p(D|H)*, and one false-alarm rate, *p*(*D*|–*H*), but there are, both for *H* and for –*H*, as many likelihoods as there are categories for the data: *p(D*_1_*|H), p(D*_2_*|H)*,…, *p*(*D*_*n*_|*H*) and *p(D*_1_*|* –*H), p(D*_2_*|* –*H)*,…, *p*(*D*_*n*_|–*H*), respectively. Correspondingly, there are as many posterior probabilities (with their complements) as there are data categories: *p(H|D*_1_*), p(H|D*_2_*)*,…, *p(H|D*_*n*_*)*. Figure 1B, illustrates a situation used in the studies reported below, namely a cue that has either a positive, a negative, or an unknown value.

Figure 1C depicts a situation with three hypotheses. For instance, a fever may have many different causes, so no physician will prescribe a drug on the basis of fever alone but will ask further questions to assess the probabilities for multiple candidate reasons. Accordingly, while there are two likelihoods in the basic task—the hit rate, *p(D|H)*, and the false-alarm rate, *p*(*D*|–*H*)—there are now as many conditional probabilities for the complex task as there are hypotheses: *p(D|H*_1_*), p(D|H*_2_*)*,…, *p*(*D*|*H*_*m*_). The same applies for the posterior probability, where there are no longer just two, *p(H|D)* and *p(*–*H|D)*, but rather as many probabilities as there are hypotheses, *p(H*_1_*|D), p(H*_2_*|D)*,…, *p(H*_*m*_*|D)*.

Finally, Figures 1D,E depict a situation with more than one cue. Asking for more information after the doctor has learned that the patient has fever amounts to inspecting more cues or performing additional tests.

How do natural frequencies affect Bayesian performance in these three complex tasks? Whereas Gigerenzer and Hoffrage (1995) left open whether the beneficial effect of natural frequencies can be generalized to more complex tasks, Massaro (1998) questioned, as mentioned before, this possibility for situations with more than one cue. Unlike in Figure 1D, he did not add one layer per cue but instead arranged the possible combinations of cue values—for a situation with two cues—in one single layer. That is, directly under the node depicting that “hypothesis *H* is true,” he placed four branches depicting the four possible combinations of two dichotomous cues: +*C*_1_&+*C*_2_, +*C*_1_&–*C*_2_, –*C*_1_&+*C*_2_, and –*C*_1_&–*C*_2_ (where + and – denote positive and negative cue values for the two cues *C*_1_and *C*_2_). Moreover, he argued that “it might not be reasonable to assume that people can maintain exemplars of all possible symptom configurations” (p. 178). However, he did not provide any empirical evidence for this claim. We fill this gap by analyzing how participants perform in complex Bayesian tasks dependent on whether information is provided in terms of probabilities or natural frequencies.

## Study 1: bayesian inferences in complex tasks

### Method

Participants were advanced medical students (*N* = 64) of the Free University of Berlin. Each of them was asked to work on four medical diagnostic tasks. Task 1 was a Bayesian task corresponding to Figure 1B, in which we extended Eddy's mammography task by adding unclear test results. Task 2 was a Bayesian task corresponding to Figure 1C, where a test could detect two diseases, namely Hepatitis A and Hepatitis B. Tasks 3 and 4 were Bayesian tasks with two and three cues, corresponding to Figure 1D and Figure 1E, respectively. In Task 3, breast cancer had to be diagnosed based on a mammogram and an ultrasound test. In Task 4, an unnamed disease had to be diagnosed on the basis of three medical tests, simply named Test 1, Test 2, and Test 3. The participants could work on the four tasks at their own pace, which took them, on average, about 1 h in total.

Each participant received the statistical information for two of the four tasks in probabilities and the other two in natural frequencies. As an illustration, Table 1 displays the two different versions (probability version vs. natural frequency version) of Task 3. The exact formulations of Tasks 1, 2, and 4 can be seen in Appendix I (Supplementary Material). Note that for Tasks 3 and 4, not all natural frequencies on the lowest layer (i.e., for all combinations of the two and three cues, respectively) were stated, but only those for which all tests were positive. Besides requesting a numerical answer to each of these four tasks, we also asked the participants to make notes and to justify their answers so that we could better understand their reasoning processes. Pocket calculators were not allowed. Following Gigerenzer and Hoffrage (1995), we classified a response as Bayesian if it was either the exact Bayesian solution or rounded to the next full percentage point.

**Table 1:** **Study 1, Task 3: A generalization of the basic Bayesian task to a more complex task with two cues (corresponding to Figure 1D)**.

**Probability version**	**Natural frequency version**
The probability of breast cancer is 1% for a woman at age 40 who participates in routine screening. If a woman has breast cancer, the probability is 80% that she will have a positive mammogram. If a woman does not have breast cancer, the probability is 9.6% that she will also have a positive mammogram. If a woman has breast cancer, the probability is 95% that she will have a positive ultrasound test. If a woman does not have breast cancer, the probability is 4% that she will also have a positive ultrasound test.	100 out of every 10,000 women at age 40 who participate in routine screening have breast cancer. 80 out of every 100 women with breast cancer will receive a positive mammogram. 950 out of every 9900 women without breast cancer will also receive a positive mammogram. 76 out of 80 women who had a positive mammogram and have cancer also have a positive ultrasound test. 38 out of 950 women who had a positive mammogram, although they do not have cancer, also have a positive ultrasound test.
What is the probability that a woman at age 40 who participates in routine screening has breast cancer, given that she has a positive mammogram and a positive ultrasound test?	How many of the women who receive a positive mammogram and a positive ultrasound test do you expect to actually have breast cancer?

**Figure 2:**
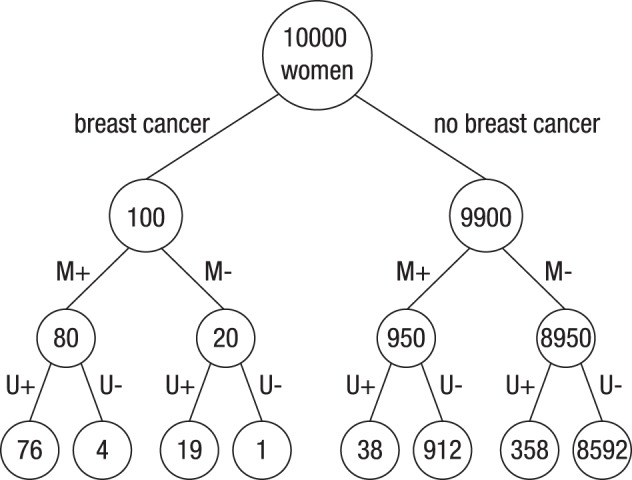
**Visual representation of the information provided in the natural frequency version of Task 3 of Study 1**. “M” and “U” denote mammography and ultrasound Test, and “+” and “−” denote positive and negative test results, respectively.

Figure 2 illustrates the frequency tree for the information provided in Task 3. Note, however, that the participants in Study 1 were neither presented with trees nor told to construct them; rather, they had to solve the task based on the wording alone^1^.

### Results

**Figure 3:**
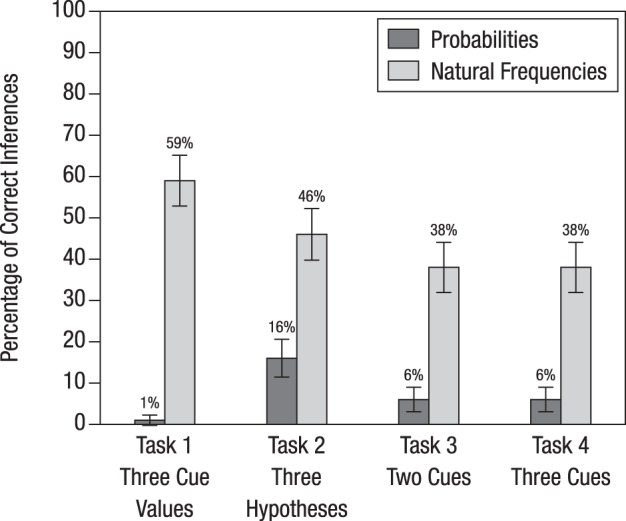
**Percentage of correct inferences in the four tasks used in Study 1**. The bars display standard errors.

Figure 3 displays the percentage of correct Bayesian inferences for each of the four tasks. In all of the tasks, replacing probabilities with natural frequencies helped the medical students make better inferences. The percentage of correct Bayesian inferences averaged across the probability versions of the four tasks was 7%; across the natural frequency versions it was 45%. Natural frequencies were most effective in Task 1, where the difference in terms of participants' performance between the natural frequency and the probability version was 59% – 1% = 58 percentage points. In the other three tasks, the increase in participants' performance from the probability versions to the natural frequency versions was about 30 percentage points. A comparison of Tasks 3 and 4 suggests that, for both the probability and the natural frequency versions, it did not matter whether information was provided on two or on three cues or whether this information referred to named or unnamed tests and diseases.

### Discussion

Study 1 showed that natural frequencies facilitate Bayesian reasoning in four complex tasks, relative to probabilities. How does the effect of natural frequencies on solving complex tasks compare to their effect on solving a basic task? One might expect that Bayesian performance in complex tasks decreases in both formats and—due to bottom effects for the probability format—that the facilitating effect of natural frequencies is less pronounced for complex tasks. However, that does not seem to be the case. Both in the present Study 1 and in Gigerenzer and Hoffrage (1995, Study 1), who used the same kind of problems, albeit for basic tasks, the average increase in performance when given natural frequencies rather than probabilities was similar, 38 percentage points in the present study (averaged across the 4 tasks) and 30 percentage points in their study. Thus, the comparison between these studies suggests the surprising conclusion that increased complexity may not decrease the effect of natural frequencies much. Whether that also holds for levels of complexity that go beyond those studied here is unknown.

## Study 2: transfer learning

Sedlmeier and Gigerenzer (2001) and Kurzenhäuser and Hoffrage (2002) have shown that the beneficial effect of presenting information in natural frequencies can be enhanced by teaching people to use this representation. In one of their studies, Sedlmeier and Gigerenzer gave two groups of participants a computerized tutorial: One group was taught how to represent probabilities in terms of natural frequencies, supported by two visual aids—frequency grid and frequency tree (representation training); the other was taught Bayes' rule for probabilities (rule training). After training, participants in each group were tested on tasks in which the statistical information was always provided in terms of probabilities. The immediate learning success for the representation training group was an improvement from 10 to 90% Bayesian answers, compared to an improvement from 0% to about 65% for the rule training group. More important, the improvement in the representation training condition was stable over time. Even 5 weeks after training, the performance of the participants who had learned to use natural frequencies remained a high 90%, whereas the performance of the group with rule training dropped to about 20%. These results were obtained for basic Bayesian tasks.

In Study 2 we addressed the question of whether in place of a computerized training program, a simple written instruction on how to solve a basic task could improve participants' ability to solve complex tasks. Extending Study 1, which investigated whether the beneficial effect of presenting information in terms of natural frequencies could also be observed for complex Bayesian tasks, Study 2 investigated whether the beneficial effect of teaching Bayesian reasoning by training representations with a basic task can also be observed when participants are later tested with complex Bayesian tasks (for which they did not receive any training).

### Method

We recruited advanced medical students (*N* = 78) from Berlin universities (none of them was a participant in Study 1). In the first step, each participant received a two-page instruction sheet on how to solve the mammography task, that is, a basic task with two hypotheses and one dichotomous cue. There were three different instructions, and participants were randomly assigned to one of them [all three instructions are shown in Appendix II (Supplementary Material)]. For Group 1, the mammography task was presented in terms of probabilities, and participants were shown how they could solve it by inserting the probabilities into Bayes' rule. For Group 2, the mammography task was presented in terms of probabilities, but here participants were instructed how to translate the probabilities into natural frequencies, how to place these frequencies into a tree, and how to determine the answer from this tree. For Group 3, the mammography task was presented in terms of natural frequencies (but no probabilities were provided), and these participants also received instructions on how to solve it by means of the frequency tree.

After studying their instruction sheet, participants were given two test tasks—the same that were used in Task 1 (one cue with three cue values) and Task 3 (two cues with two cue values each) in Study 1. Participants of Group 1 and 2 received probability versions of these tasks, and participants of Group 3 received the natural frequency version. The instruction sheet was at their disposal while working on the complex tasks. Table 2 summarizes the design of Study 2.

**Table 2:** **Experimental design in Study 2: Three ways to instruct participants to solve the mammography task**.

	**Group 1 (*N* = 27)**	**Group 2 (*N* = 25)**	**Group 3 (*N* = 26)**
Basic task used for instruction	Mammography task, formulated in terms of probabilities	Mammography task, formulated in terms of probabilities	Mammography task, formulated in terms of natural frequencies
Solution explained in instruction	How to insert probabilities into Bayes' rule	(a) How to translate probabilities into natural frequencies	How to place these natural frequencies into a frequency tree and to extract the correct answer
		(b) How to place these natural frequencies into a frequency tree and to extract the correct answer	
Complex tasks tested	Tasks 1 and 3 of Study 1 (both tasks in probabilities)	Tasks 1 and 3 of Study 1 (both tasks in probabilities)	Tasks 1 and 3 of Study 1 (both tasks in natural frequencies)

*For details, see Appendix II in Supplementary Marerial*.

### Results

**Figure 4:**
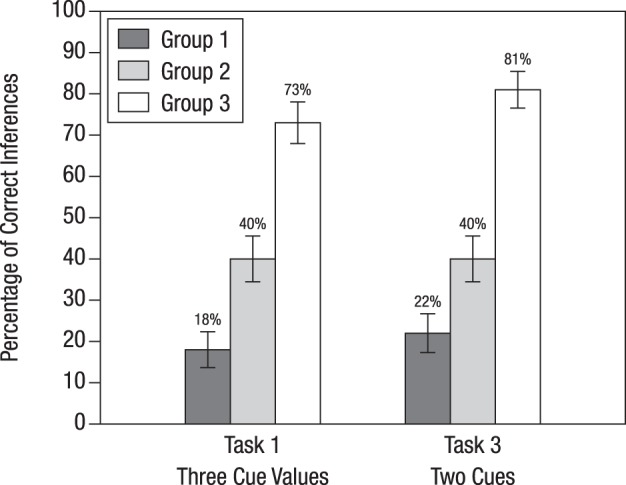
**Percentage of correct inferences for Tasks 1 and 3, depending on how participants were instructed to solve Bayesian inference tasks in Study 2**. The bars display standard errors. Tasks 1 and 3 are the same test tasks as those that were used for Study 1. The three instruction conditions are summarized in Table 2 and can be seen in full length in Appendix II (Supplementary Material).

Figure 4 displays the percentages of Bayesian inferences in Tasks 1 and 3 separately for the three experimental groups. In both tasks, participants' performance was about the same, which suggests that the differences found in Study 1 disappear when there is an instruction on the basic task.

For the basic task, participants in Group 1 learned how to insert probabilities into Bayes' rule. Then they were tested on whether this training generalizes to applying Bayes' rule to more complex tasks in which information is presented in probabilities. Compared to Groups 2 and 3, this group performed worst when confronted with complex Bayesian tasks (18% for Task 1 and 22% for Task 3). Nonetheless, their percentage of Bayesian inferences was substantially higher compared to that of participants of Study 1 for the same tasks (1% for Task 1 and 6% for Task 3; see Figure 3). Hence, we can conclude that the instruction had a positive effect: At least some of the participants managed to extend Bayes' rule to a more complex task involving an unclear test result (which amounts to adding a corresponding term to the denominator of Equation 2) and to a more complex task involving the results of two different tests (which amounts to applying Bayes' rule twice, that is, first computing the posterior probability after the first test result became known, and then using this probability as a prior probability to compute the posterior probability after the result of the second test became known).

Participants in Group 2 had learned, for the basic task, how to translate probabilities into natural frequencies. In spite of also being tested on tasks with information presented in terms of probabilities, 40% of participants in Group 2 obtained the correct solutions (this percentage happened to be identical for Tasks 1 and 3). These participants arrived at these solutions by performing the following steps: First, they correctly translated five probabilities (rather than three, as was the case for the basic task) into natural frequencies. To construct a corresponding tree they added nodes to the tree they had seen in the instruction. For Task 1 they had to add two nodes on the lowest layer (as can be seen when comparing Figure 1A and Figure 1B), and for Task 3 they had to add an additional layer for the outcomes of the ultrasound test (as can be seen when comparing Figure 1A and Figure 1D). From these modified trees they finally extracted the frequencies needed for the Bayesian solutions in the form of “Laplacian proportions,” that is, the ratio of relevant cases divided by the total number of cases.

The participants of Group 3 were the only ones who were trained and tested with natural frequencies. This instruction method led to a high performance rate of 73% (Task 1) and 81% (Task 3). In contrast to Group 2, participants of Group 3 only needed to extend frequency trees; no translation of probabilities into frequencies was required. Recall that without prior instruction on the basic task, performance on the same two tasks was lower, 59 and 38%, respectively (Study 1). When comparing the performance gain for Task 1 (from 59% in Study 1, without instruction, to 73% in Study 2, with instruction) with the corresponding performance gain for Task 3 (a rise from 38 to 81%), it becomes obvious that instructions based on frequency representations affected the two types of generalizations differentially. Analyzing participants' protocols confirmed this pattern: Participants found it easier to take the tree from the basic task and to add another layer than to add nodes within a layer. In other words, generalizing the basic task (Figure 1A) to Task 3 (Figure 1D) seemed to be more intuitive for the participants than generalizing it to Task 1 (Figure 1B).

### Discussion

Previous studies have established the usefulness of teaching how to represent probability information in terms of natural frequencies (Kurzenhäuser and Hoffrage, 2002; Sedlmeier and Gigerenzer, 2001; Ruscio, 2003; Sirota et al., 2015a). Study 2 extends these findings by showing that a simple instruction on how to solve a basic Bayesian task can amplify performance in complex tasks. The highest levels were obtained when both the trained task and the tested task were consistently formulated in terms of natural frequencies. That is, it is largely sufficient to instruct people in using natural frequencies in the basic task in order to ensure a generalization to and solution of complex tasks, as long as the information in both cases is in natural frequencies.

## General discussion

This paper has two results, one conceptual and one empirical. Figure 1 shows how the natural frequency tree for the basic task (Figure 1A) can be generalized to various complex Bayesian tasks. As these trees (displayed in Figures 1B–E) demonstrate, the possibility of communicating statistical information in terms of natural frequencies is not restricted to the basic task with one dichotomous cue for inferring which of two hypotheses is true. Being able to generalize from these trees is important because in many real-life situations such as medical diagnosis or court trials, information is not dichotomous, several (rather than only one) pieces of evidence are available, and/or more than two hypotheses are considered.

With Study 1, we have empirically shown that, despite the trees for complex tasks having more branches than in the tree for the basic task, the facilitating effect of natural frequencies is essentially in the same order of magnitude as in previous studies using the basic task. Study 2 showed that instructing people how to use natural frequencies to solve the basic task was helpful for solving complex Bayesian tasks. Apparently, the best method is to instruct directly how to reason with natural frequencies and also to test people on natural frequencies. Instruction adds to the mere effect of representation demonstrated in Study 1. In contrast to claims made in the literature (Massaro, 1998), each of our studies show that the power of natural frequencies generalizes to complex tasks. In the remainder of this paper, we will discuss the power (and limits) of natural frequencies and that of instructions.

## Power (and limits) of natural frequencies in complex tasks

This study has shown that the natural frequency approach to Bayesian reasoning is powerful enough to be generalized to complex tasks and to allow for good performance despite increasing numbers of cues and cue values. How do natural frequencies support reasoning? Gigerenzer and Hoffrage (1995) demonstrated in detail that natural frequencies reduce the number of computational steps necessary for Bayesian inference and derived seven specific results, including that relative frequencies do not simplify the computation. Subsequent work has used different terms for the same explanation: the subset principle, set inclusion, or the nested-set hypothesis (for a discussion of these terms and their relationship to natural frequencies, see Hoffrage et al., 2002; Brase, 2007; Ayal and Beyth-Marom, 2014). Moreover, Ayal and Beyth-Marom also quantified the computational simplification and counted the mental steps or elementary information processes as a measure of the cognitive effort required to complete the task (for a similar analysis, see Johnson and Tubau, 2015).

Extending this analysis to complex tasks is straightforward and reveals that natural frequencies require less cognitive effort not only for basic tasks but also for complex tasks. However, even natural frequencies require computation and effort. Hence it does not come as a surprise (1) that for tasks using natural frequencies, the proportion of Bayesian inferences is less than 100% and (2) that variables related to participants' computational abilities can account for variance in Bayesian performance. For instance, performance in Bayesian inference tasks—both for probability and natural frequency representations—is correlated with numeracy (Chapman and Liu, 2009; Johnson and Tubau, 2015), numerical skills (Tubau, 2008), and fluid cognitive ability and thinking disposition (Sirota et al., 2014) (for a discussion of individual differences in Bayesian reasoning, see Brase and Hill, 2015).

At the same time, natural frequency representations have their limits. As mentioned earlier, Massaro (1998) argued that “a frequency algorithm will not work” because “it might not be reasonable to assume that people can maintain exemplars of all possible symptom configurations” (p. 178). We have meanwhile seen that in a textbook task, half (and with instruction, three quarters) of our participants were able to process the statistical properties of three cues in a Bayesian way when this information was represented in terms of natural frequencies. Notwithstanding this result, we share Massaro's concern that at some point humans are no longer able to store the frequencies for all possible conjunctions of cues in memory. In fact, in a situation with 10 dichotomous cues, the corresponding frequency tree would carry 2048 natural frequencies on the lowest layer, and with 20 cues this number would be over 2 million. It may nonetheless be possible to learn the statistical relationships between hypotheses and cues as the number of cues grow larger—after all, for two hypotheses and a dichotomous cue there are only four proportions (or probabilities) that are relevant and need to be learned: *p(D|H), p*(*D*|–*H*), *p(H*|*D), p*(*H|*–*D)*. Learning the statistical relationships for *conjunctions* of cues, however, is a huge challenge because the number of relevant proportions would no longer grow linearly with the number of cues (four per cue) but instead exponentially.

As the number of cues grows larger, the difference between real-life settings and textbook tasks becomes increasingly important. Whereas it is difficult, if not impossible, to memorize and manage the relevant information in a real-life setting, which corresponds to a probability learning paradigm, it is possible to represent the natural frequencies required for Bayesian inferences in a textbook task. But even natural frequency representations in textbook tasks have their limits. These may not yet be reached for three cues, as our empirical findings reported above suggest, but draw nearer as the tree grows larger. The two major limits are practical feasibility and robustness. First, practical feasibility is hampered by the sheer amount of information that needs to be communicated—recall that a frequency tree for two hypotheses and 10 (20) dichotomous cues would have 2048 (>2,000,000) natural frequencies on the lowest layer. Second, and relatedly, for many real-life applications the number of observations for a particular combination of cues will most likely be relatively small. Because of the resulting estimation error, the Bayesian inferences may have fairly wide confidence intervals and may thus not be very robust.

## Fast-and-frugal trees

What tools remain for the boundedly rational human mind (and for animals) in complex situations with a vast number of cues? We assume that the human mind is equipped with an adaptive toolbox containing simple heuristics that allow “fast-and-frugal” decisions, even in highly complex environments (Gigerenzer et al., 1999, 2011; Gigerenzer and Selten, 2001; Todd et al., 2012; Hertwig et al., 2013). These simple heuristics are helpful when making inferences in situations under limited time, with limited knowledge, and within our cognitive and computational constraints. One of the characteristics of these simple heuristics is that they reduce information intake and processing. Complexity—and note that this is the direction in which we extended the basic task—can be reduced tremendously by assuming conditional independence between cues, which is exactly what participants seem to do unless they have strong evidence speaking against this assumption (Waldmann and Martignon, 1998; Martignon and Krauss, 2003). To the extent that this assumption is justified, it is no longer necessary to store the millions of possible conjunctions of 20 dichotomous cues in memory, but it would be sufficient to represent the predictive power of a cue independent of the other cues.

**Figure 5:**
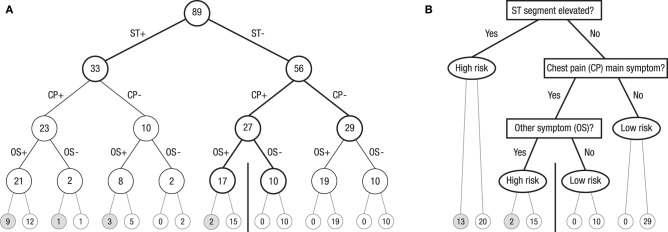
**(A)** Full natural frequency tree for the Green and Mehr (1997) data on 89 patients with severe chest pain. The goal is to determine whether these patients are at high or low risk for heart disease. ST denotes a particular pattern in the electro cardiogram, CP denotes chest pain, OS denotes “at least one other symptom,” “+” denotes present, and “–” denotes absent. Numbers in circles denote number of patients. **(B)** Fast-and-frugal classification tree obtained by pruning the natural frequency tree. The ranking of cues and the exit structure are determined by the ZigZag method (in the present case, ZigZag-val and ZigZag-sens, as explained in the text, lead to the same trees). Questions in rectangles specify which cues are looked up at this level for each of the patients in the corresponding circles in **(A)**. Depending on whether this cue value is positive or negative, either a new question is asked or the tree in **(B)** is exited and a classification decision is made (oval). The accuracy of these classification decisions is shown by the number of patients below these oval exit nodes: The number of patients who actually had a heart attack is displayed in the left of the two adjacent end nodes in the lowest layer, and the number of those who did not have one is displayed in the corresponding end node on the right. All patients to the left of the vertical bar in Figure 1B are classified as high risk, and all patients to its right are classified as low risk.

The reduction of complexity can be achieved in many ways. Radically pruning a natural frequency tree for many cues while maintaining all cue information converts it into a so called *fast-and-frugal tree*—which is one of the heuristics analyzed by the Center for Adaptive Behavior and Cognition at the Max-Planck Institute for Human Development in Berlin (Martignon et al., 2003). Figure 5 shows an example of such a classification tree, based on Green and Mehr (1997), for classifying patients as at high or low risk for heart disease. In Figure 5A, the full natural frequency tree for three cues is exhibited. Note that this tree displays the hypotheses (high risk vs. low risk of heart attack) no longer at the second layer, as the trees in Figure 1 do, but at the very lowest layer. Whereas the trees in Figure 1 are the usual natural frequency trees that communicate data given a hypothesis, the tree in Figure 5A displays natural frequencies *after* Bayesian updating, which, in turn, enables the classification of patients based on symptoms. Note that the trees in Figure 1 and Figure 5 carry natural frequencies (for a direct comparison of these two forms of grouping a given set of natural frequencies, see Hoffrage et al., 2015, Figures 1B,C).

The tree in Figure 5A can be radically pruned. The resulting fast-and-frugal tree, exhibited in Figure 5B, is “fast and frugal” according to the definition given in Martignon et al. (2008): At each node of the tree, the choice is either to stop further information acquisition and make a diagnosis or to collect more information. Specifically, in a first step, all 89 patients are checked for elevated ST segment in their electrocardiogram. If the answer is positive (ST+), they (*n* = 33) are classified as high risk, without considering any further information. The remaining 56 patients are checked for chest pain as the main symptom. If the answer is no (CP–), they (*n* = 29) are classified as low risk. The remaining 27 patients are checked for whether any other symptom is present. If the answer is yes (OS+), they (*n* = 17) are classified as high risk; the others (OS–) are classified as low risk (*n* = 10). Each tree level corresponds to one cue, and the ranking of cues can follow simple heuristic procedures. Green and Mehr reported that diagnosis according to this fast-and-frugal tree was more accurate than both physicians' clinical judgment and logistic regression.

Two important features of the construction of a fast-and-frugal tree are the ranking of cues and its exit structure, that is, whether an exit is to the left or to the right (with the convention that branches defined by positive cue values will always be displayed at the left). One possible ranking, called the ZigZag-val method, is achieved by using the predictive values of the cues. The *positive predictive value* of a cue is the proportion of cases with a positive outcome among all cases with a positive cue value [i.e., *p*(*H|D*)] and the *negative predictive value* is the proportion of cases with a negative outcome among all cases with a negative cue value [i.e., *p*(–*H|* –*D*)]. The ZigZag-val tree has a left exit for levels 1 to k, where k is the smallest natural number so that 1/2^*k*^ is less than the ratio of the base rate of the disease divided by the base rate of healthy patients. For the levels after the kth level, the tree alternates between “yes” and “no” exits at each level, and a choice is made according to the cue with the greatest positive (for “yes”) or negative (for “no”) predictive value among the remaining cues (Martignon et al., 2008). A second method for tree construction, ZigZag-sens, has a left exit for levels 1 to k. For the levels after the kth level, the tree alternates between “yes” and “no” exits at each level, and a choice is made according to the cue with the greatest positive sensitivity [i.e., the greatest *p(D|H)*] or specificity [i.e., the greatest *p(*–*D|* –*H)*] among the remaining ones. Ties in the process are broken randomly.

Fast-and-frugal trees—those ranked according to positive and negative predictive value or according to sensitivity and specificity—radically reduce the complexity of full natural frequency trees. Their performance can be impressive. In the tree displayed in Figure 5, the lowest layer in Figure 5A displays the number of patients who after classification actually had a heart attack (left end nodes) and those who did not (the corresponding end nodes to the right). The vertical bar in the lowest layer can be seen as cutoff. The fast-and-frugal tree in Figure 5B is arranged so that all nodes to its left (*n* = 50) are classified as high risk (yielding 15 hits and 35 false alarms), and every one of the 39 cases to right of the bar are classified as low risk (yielding 0 misses and 39 correct rejections). In particular, fast-and-frugal trees ranked by sensitivity and specificity yield ROC curves with large areas underneath. Such properties are fundamental for medical doctors to reduce costly errors, in particular, misses (for ROC curves and fast-and-frugal trees, see Luan et al., 2011).

Another class of trees that reduce complexity is that based on CART (Breiman et al., 1984); these trees are simple in execution but often require complicated computations for their construction. To reduce complexity while maintaining the tenets of the Bayesian attitude, the strategy is to adopt the Naïve Bayes approach. Its simplification consists of assuming that cues are independent conditional on presence or absence of the disease, so that the probability of disease given cues can be estimated as the product of the conditional probabilities of disease given each one of the cues.

However, the tradition among practitioners has been to make use of classification strategies based on some type of regression. For binary classification, logistic regression is the standard model used by practitioners. When using logistic regression one assigns a value of 0 to the “low” state of *H*_*k*_ and a value of 1 to the “high” state of *H*_*k*_. The logistic regression equation is:

(4) $$\frac{p\left(D|H_{1},\ldots ,H_{n}\right)}{1-p\left(D|H_{1},\ldots ,H_{n}\right)}=e^{\beta_{0}+\sum_{k}\beta_{k}H_{k}}$$

where the parameters are typically estimated from data.

Laskey and Martignon (2014) compared the predictive accuracy of these five classification methods using 11 data sets taken from medical domains. When the models were constructed based on 90% of the data set, Naïve Bayes performed best, achieving 80% accuracy, while Logistic Regression achieved 79%. CART, like the ZigZag-val tree, achieved 74% accuracy, while the ZigZag-sens tree achieved 72% accuracy (note that in Laskey and Martignon, ZigZag-val is labeled ZigZag tree and ZigZag-sens was computed but not reported). When the models were constructed based on 50% of the data, CART, ZigZag-val, and ZigZag-sens performed at the same level as when being fitted to 90% of the data, whereas Logistic Regression and Naïve Bayes lost one percentage point each. Even more surprising, when the training set amounted to only 15% of the data set, ZigZag-val outperformed logistic regression and CART. In an uncertain world, where large numbers of correlations need to be estimated, fast-and-frugal trees can reduce estimation error and can have a competitive advantage over more complex strategies, in particular for small learning samples (Luan et al., 2011).

Predictive accuracy is not the only important criterion in medical diagnosis. It is often essential to make a diagnosis quickly or with limited diagnostic information. All in all, fast-and-frugal trees make it possible to act on limited information, and by reducing estimation error, they can perform competitively in situations entailing high complexity and uncertainty. They accomplish this by inverting natural frequency trees, so that the outcome (or hypothesis) is no longer displayed at the top of the tree (as in Figure 1) but at the lowest layer (as in Figure 5A). Subsequently, they can be pruned by cutting off branches, that is, by introducing an exit at every layer of the tree (as in Figure 5B).

## Cue merging

We will now discuss another way of reducing tree complexity, which amounts to merging multiple cues into one single cue. It has been studied in a probability learning task by Garcia-Retamero et al. (2007a) and Garcia-Retamero et al. (2007b). Participants had to make pair comparisons based on three cues, C_1_, C_2_, and C_3_, with a validity (i.e., proportion of correct inferences) of 80, 60, and 60%, respectively. The cues were *not* independent. Specifically, although the cues C_2_ and C_3_ had a relatively low validity, they could be merged—by applying simple Boolean algebra—into one cue that had a validity of 100%. For instance, if C_2_ AND C_3_ was present, then the alternative to which the cue pointed was correct in 100% of the cases (in two other conditions, we constructed environments in which merging two cues with the OR combination and the XOR combination created a new cue with a validity of 100% as well). Participants were not informed about this structure, but they were told that the three cues represent whether some drugs have been given to two patients. Their task was to predict which of two patients had the higher blood pressure. In these studies, the mental models of the participants were manipulated. In one condition, participants were informed that the three drugs operate in three different systems (hormonal, nervous, blood) and in the other condition that they operated within the same system. Those participants who had been told that the three drugs operated via different systems assumed independence and did *not* detect the hidden cue structure. By contrast, a majority of those participants who had been informed that the drugs operated via the same system could not safely exclude independence and *did* detect the structure. In a mouselab task, they immediately clicked C_2_ and C_3_, inspected both values, and only if the merged cue was not present did they request C_1_ (note that they started with C_2_ and C_3_ even though each of these had a lower validity than C_1_).

As this study demonstrates, participants assume independence by default but can detect dependencies if these exist. Such detection is easy with a natural frequency representation, which obviously can be constructed even in a probability learning task. Once participants have learned that cues can be merged, they treat this new cue as a single one, even though it is composed of two (similar to the term *bachelor*, which requires the presence of two features, male and unmarried). This empirical demonstration brings to mind Green and Mehr's (1997) fast-and-frugal tree, in which one of the nodes also contains a merged cue—in that case, an OR conjunction of five cues (labeled “other symptom”; Figure 5B).

The common denominator between fast-and-frugal trees and cue merging is that both can simplify the structure of a complex natural frequency tree. Both exploit certain structures of information (such as conditional dependence) and are “ecologically rational” if these structures are present. Constructing fast-and-frugal trees amounts to inverting complex natural frequency trees (with a hypothesis at the top layer) into simple classification trees (with data at the top) that implement one-reason decision making. Such trees perform well if some cues are so informative that less predictive cues no longer add substantial predictive value and can hence be ignored. Cue merging amounts to combining several cues into one; these merged cues can lead to better inferences than any of the single cues used separately. In general, fast-and-frugal heuristics—including fast-and-frugal trees and simple heuristics for pair comparison, with or without merged cues—are ecologically rational if they are adapted to the structure of information in the environment (Martignon and Hoffrage, 1999, 2002; Todd et al., 2012). Future research has to address the question of what the crucial variables (e.g., number of cues) are that trigger switching from being a Bayesian to being fast and frugal. For a first step in this direction, see Martignon and Krauss (2003), and for an exploration of Bayesian inferences as a function of task characteristics, see Hafenbrädl and Hoffrage (2015).

## The effect of natural frequencies can be amplified by visual representations

In Study 2, we used natural frequencies to instruct participants how to reason the Bayesian way. In this context, we also presented the frequency tree to participants (see Appendix II in Supplementary Material). Such a tree supports any text in explaining natural frequencies through a visualization of the information structure relevant to solve a Bayesian inference task. But trees are not the only tool that can serve this function. Others are icon arrays, Euler diagrams, frequency grids, unit squares, and roulette wheel diagrams (for an overview see Binder et al., 2015; Mandel, 2015). Garcia-Retamero and Hoffrage (2013) demonstrated that patients' performance in a basic Bayesian inference task could be improved through a frequency grid whose effect is above and beyond that of natural frequency representation in the written text. The most common visualizations used in teaching statistics in schools, however, tend to be 2 × 2 tables and tree diagrams, both of which explicitly contain numbers. Note that these visual aids can make use of natural frequencies or probabilities and improve participants' performance when natural frequencies are used: In a study by Steckelberg et al. (2004), the beneficial effect of natural frequencies was about the same in both conditions. By contrast, tree diagrams and 2 × 2 tables using probabilities (or relative frequencies) do not improve participants' performance—yet are omnipresent in textbooks on probability theory (for an empirical study on the effect of these visualizations beyond pure format effects, see Binder et al., 2015).

With respect to visualization of Bayesian reasoning situations with two hypotheses and more than two cue values, both trees and tables can be easily extended to illustrate such situations (e.g., for three cue values, see the tree in Figure 1B, and imagine a 2 × 3 table). Likewise, a situation with more than two hypotheses and a dichotomous cue can easily be represented by a tree (e.g., for three hypotheses, see the tree in Figure 1C, and imagine a 3 × 2 table). However, situations with more than two cues appear to be easier to represent by trees (e.g., Figure 1D) than by tables. The ease of constructing and generalizing tree diagrams containing natural frequencies was the reason for choosing this visual aid in Study 2.

All in all, the available evidence shows that natural frequencies can facilitate Bayesian reasoning in situations of risk, that is, where probabilities are (assumed to be) known, as in textbook problems. The novel insights of this article are that this power extends to complex Bayesian tasks and that teaching natural frequencies in basic tasks generalizes to complex tasks. These insights correct the widespread claim that people are not built to reason the Bayesian way, and, more important, they provide an efficient tool to teach Bayesian reasoning even in complex situations.

## Author note

The studies reported in this manuscript were approved by the ethic committee of the Max Planck Institute for Human Development, Berlin, and were carried out with written informed consent from all participants. We would like to thank the three reviewers and the editors for their valuable feedback and Rona Unrau for editing the manuscript. This work was supported by grant 100014_140503 from the Swiss National Science Foundation.

### Conflict of interest statement

The authors declare that the research was conducted in the absence of any commercial or financial relationships that could be construed as a potential conflict of interest.

## Footnotes

^1^Note that the wording of the probability version of Task 3 is mute on the question of whether or not the two tests are independent of each other. Even though each of the two tests is dependent on the disease, they are indeed independent of each other for any level of the variable *disease*, and we anticipated that our participants (advanced medical students) knew this. An analysis of the participants' protocols revealed that all of them implicitly made this assumption. Participants' intuitive assumption of independence was also found in Task 4, where information on three unnamed tests was provided. This finding is in accordance with the finding of Waldmann and Martignon (1998) that people assume conditional independence between cues as long as there is no explicit evidence suggesting dependency.
