# Supplementary material for: Natural frequencies improve Bayesian reasoning in simple and complex inference tasks
Source: Front Psychol. 2015 Oct 14;6:1473. doi: 10.3389/fpsyg.2015.01473 (PMC4604268; doi:10.3389/fpsyg.2015.01473)
Supplement: Supplementary file 1 [file DataSheet1.DOCX]

## Appendix I:

Study 1: Probability and Natural Frequency Versions of Tasks 1–4 (Tasks 1 and 3 were also used in Study 2)

Task 1: Three Cue Values (positive, negative, and unclear test result)

| Probability Version  The probability of breast cancer is 13.8% for a woman with a dominant gene mutation.  If a woman with a dominant gene mutation actually has breast cancer, the probability is:  - 53.7% that she will receive a positive mammogram,  - 24.6% that she will receive an unclear result, and  - 21.7% that she will receive a false negative mammogram.  If a woman with a dominant gene mutation does not have breast cancer, the probability is:  - 7.7% that she will receive a positive mammogram,  - 7.7 % that she will receive an unclear result, and  - 84.6% that she will receive a false negative mammogram.  What is the probability that a woman with a dominant gene mutation actually has breast cancer, given that she has a positive mammogram? | Natural Frequency Version  138 out of every 1,000 women with a dominant gene mutation have breast cancer.  Out of every 138 women with a dominant gene mutation who actually have breast cancer:  - 80 will receive a positive mammogram,  - 34 will receive an unclear result, and  - 30 will receive a false negative mammogram.  Out of every 862 women with a dominant gene mutation who do not have breast cancer:  - 66 will receive a positive mammogram,  - 66 will receive an unclear result, and  - 730 will receive a false negative mammogram.  How many of the women with a dominant gene mutation who receive a positive mammogram do you expect to actually have breast cancer? |
| --- | --- |

Task 2: Three Hypotheses (disease A, disease B, or healthy)

| Probability Version  The probability of having disease A is 0.4% for a person without any symptoms.  The probability of having disease B is 0.1% for a person without any symptoms.  (Diseases A and B never occur at the same time.)  There is a medical test which can detect both diseases. But it is unable to discriminate between disease A and B and, furthermore, it is not safe.  - If a person has disease A, the probability is 90% that she or he will receive a positive result.  - If a person has disease B, the probability is 80% that she or he will receive a positive result.  - If a person has neither disease A nor disease B, the probability is 10% that she or he will nevertheless receive a positive result.  What is the probability that a person without any symptoms obtains a positive test result, if the person actually suffers from  - disease A?  - disease B?  - any of the diseases (A or B)? | Natural Frequency Version  40 out of every 10,000 persons without any symptoms have disease A and 10 have disease B.  (Diseases A and B never occur at the same time.)  There is a medical which that can detect both diseases, but it is unable to discriminate between disease A and B and, furthermore, it is not safe.  36 out of every 40 persons who have disease A receive a positive result.  8 out of every 10 persons who have disease B receive a positive result.  995 out of every 9,950 persons who do not have disease A nor B receive a positive result.  How many of the persons without any symptoms who receive a positive test result suffer from  - disease A?  - disease B?  - any of the diseases (A or B)? |
| --- | --- |

Task 3: Two Cues (two medical tests).

Task 4: Three Cues (three unnamed medical tests).

| Probability Version  The probability of having a special complaint is 2%.  If a person has the complaint, the probability is 80% that she or he will receive a positive test result in test 1.  If a person has the complaint, the probability is 95% that she or he will receive a positive test result in test 2.  If a person has the complaint, the probability is 75% that she or he will receive a positive test result in test 3.  If a person does not have the complaint, the probability is 25% that she or he will nevertheless receive a positive test result in test 1.  If a person does not have the complaint, the probability is 10% that she or he will nevertheless receive a positive test result in test 2.  If a person does not have the complaint, the probability is 20% that she or he will nevertheless receive a positive test result in test 3.  What is the probability that a person actually has the complaint if all 3 tests are positive? | Frequency Version  200 out of every 10,000 persons have a complaint.  160 out of every 200 persons who have the complaint receive a positive result in test 1.  152 out of every 160 persons who have the complaint receive a positive result in test 2.  114 out of every 152 persons who have the complaint receive a positive result in test 3.  2,450 out of every 9,800 persons who do not have the complaint nevertheless receive a positive result in test 1.  245 out of every 2,450 persons who do not have the complaint nevertheless receive a positive result in test 2.  49 out of every 245 persons who do not have the complaint nevertheless receive a positive result in test 3.  How many of the persons who receive a positive result in all 3 tests actually have the complaint? |
| --- | --- |

## Appendix II:

Instructions for the three experimental groups in Study 2.

Each group first received a brief **Introduction**, which was identical for all groups, followed by their **Training Task**. Subsequently, participants were informed about **Terminology and Solution**. These modules were partly identical for Group 1, Group 2, and Group 3. The following appendix is ordered by the three modules and, within the module, by the variants that differed between the experimental groups.

**(Introduction, identical for all groups)**

Here we present a medical diagnosis task and elucidate its mathematical solution. We are interested in whether it is possible to generalize this explanation to more complex tasks. Thus, you will receive two tasks that are structured similarly to the elucidated one but are slightly more complex. Your task is to try to solve both complex tasks by extending the provided explanation.

**(Training Task, Groups 1, 2)**

*The probability of breast cancer is 1% for a woman at age 40 who participates in routine screening. If a woman has breast cancer, the probability is 80% that she will receive a positive mammography. If a woman does not have breast cancer, the probability is 10% that she will also receive a positive mammography. A woman in this age group had a positive mammography in a routine screening. What is the probability that she actually has breast cancer?*

**(Training Task, Group 3)**

*10 out of every 1,000 women at age forty who participate in routine screening have breast cancer. 8 out of every 10 women with breast cancer will receive a positive mammography. 99 out of every 990 women without breast cancer will also receive a positive mammography. Here is a new representative sample of women at age forty who got a positive mammography in a routine screening. How many of these women do you expect to actually have breast cancer?*

**(Terminology and Solution, Group 1)**

- The first number (1%) describes the *prevalence*, which is the probability that a woman in the sample actually has breast cancer (B). Mathematically we write this as p(B) = 1%.

- The second number (80%) is the *sensitivity* of the mammography (M). The sensitivity is the probability that the mammography detects the disease if it is present. Mathematically we write p(M+ | B).

- The last number (10%) is the *false-alarm rate*. This is the probability that the mammography indicates breast cancer even though it is not present. Mathematically the false-alarm rate is p(M+ | –B).

To assess the probability that a woman has cancer when given a positive mammogram, i.e., p(B | M+), one can insert these probabilities into Bayes’ rule:

$p(B | M+)$ $= \frac{p\left( B \right)p(B|M+)}{p\left( B \right)p\left( B | M+ \right)+p\left( -B \right)p(-B| M+)}$ (2),

The probability p(–B), that is, the probability that a woman in the sample does not have breast cancer, cannot be found directly in the task description, but it can easily be deduced. It is the complement of p(B) and thus: p(–B) = 1 – p(B) = 99%. Together we have:

$p\left( B \right| M+)$ $= \frac{1\% x 80\%}{1\% x 80\% +99\% x 10\%}$ $=7.5\%$ (2),

This means: If a woman in this sample receives a positive mammogram, the probability that she indeed has breast cancer is only 7.5%.

**(Terminology and Solution, Group 2)**

- The first number (1%) describes the *prevalence*, which is the probability that a woman in the sample actually has breast cancer (B).

- The second number (80%) is the *sensitivity* of the mammography (M). The sensitivity is the probability that the mammography detects the disease if it is present.

- The last number (10%) is the *false-alarm rate*. This is the probability that the mammography indicates breast cancer even though it is not present.

To assess the probability that a woman has cancer when given a positive mammogram, it is convenient to translate the given information into frequencies and then depict these frequencies in a tree diagram.

Imagine a population of women, say, 1,000. To construct a frequency tree in a first step, the population has to be divided into ill and healthy women. The prevalence says that out of every 1,000 women, 1% have breast cancer, which is 10. Consequently, 990 women are healthy.

In a second step, both the healthy and the ill women have to be divided again according to the sensitivity (which makes a statement about the ill women) and the false-alarm rate (which makes a statement about the healthy women).

The sensitivity of 80% indicates that out of the 10 women with breast cancer, 8 will be detected by a mammography and 2 will be missed by this test. The false-alarm rate of 10% indicates that out of the 990 healthy women, 99 will be wrongly classified as having breast cancer. Now we have all numbers to yield the following tree diagram:


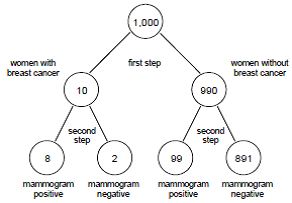


From the tree diagram, the answer to the question “How many of the women with a positive mammogram actually have breast cancer?” can be seen. Overall, 107 = 8 + 99 women obtained a positive mammogram. Out of these, only 8 actually have breast cancer. Therefore, the probability of having breast cancer provided with a positive mammogram is:

$\frac{8}{8+99} =\frac{8}{107} =$7.5%

Because we want to know how you can transfer what you learned, you will now receive two – slightly more difficult – new problems.

(Participants now were confronted with Tasks 1 and 3 (Appendix I) in terms of probabilities.)

**(Terminology and Solution, Group 3)**

- The first frequency (10 out of 1,000) is the *prevalence*, which is the proportion of women with breast cancer in the sample (of 1,000 women).

- The second frequency (8 out of 10) is the *sensitivity* of the mammography. It indicates how many of a population of women with breast cancer the mammography will detect.

- The last frequency (99 out of 990) is the *false-alarm rate*. It indicates how many of a population of healthy women will wrongly be classified as having breast cancer by the mammography.

To “see” the solution of the task it is convenient to depict these frequencies in a tree diagram, which should be developed step-by-step, beginning at the top.

In a first step, the population has to be divided into ill and healthy women. This can be calculated by looking at the prevalence.

In a second step, both the ill and the healthy women have to be divided again according to the sensitivity (which makes a statement about the ill women) and the specificity (which makes a statement about the healthy women) of the mammography.

Conducting both steps yields the following tree diagram:

(Participants of Group 3 were now presented with the same tree as that of Group 2, including the derivation of the solution. Afterwards they were given Tasks 1 and 3 (Appendix I) in terms of natural frequencies.)
